# iConsent an Electronic Consent Platform with the MS Register

**DOI:** 10.23889/ijpds.v1i1.363

**Published:** 2017-04-19

**Authors:** Rod Middleton, David Ford, Daniel Naeh

**Affiliations:** 1 Swansea University

## Objectives

The UK MS Register is a large scale observational research platform, capturing data from patients, NHS and carries out linkage with routine data from the SAIL databank.

We have 14,000 People with MS (PwMS) submitting Patient Reported Outcome Measures (PRoMS) quarterly and over 3000 patients consenting at NHS Sites around the UK

A differentiating factor between Register and previous attempts to capture PRoMS and clinical data was the goal that it be paperless. One area, where paper had to be used, was obtaining informed consent. Clinical participants are consented using a triplicate consent form, one copy for the patient, one for medical notes and one for the Register

It's desirable for patients to be able to electronically consent, providing the following benefits:

- Tablet computers already in use to collect PRoMS
- Printing costs
- Participant expectations
- Improved content and user experience: improved feedback, of multimedia elements about informed consent
- Increased familiarity with tablets

## Approach

Changing consent methodology is complex, all documentation, processes and changes are reviewed by the ethics committee. A privacy protecting, secure software package (iConsent) was developed by modifying an existing package from Welsh Cancer Bank.

The software is server based, running on a Secured MS SQLServer 2014 and developed in .net to iOS/Android tablets

The practitioner taking consent explains the process, participants then see the approved documentation and materials. Finally they fill in their email address and name, and are presented with the consent form, the participant uses a stylus to sign. The practitioner then countersigns.

Once completed a digitally signed, secure pdf is generated on the server. Links are sent by email to the participant, the Register and unit administrator. The pdf is functionally identical to the paper. 

## Results

The South West Central Bristol ethics committee approved the software following guidance on security and documentation design. Staff were trained in system usage.

A number of patients were successfully e-consented, Of note was a potential issue with some patients and how MS impacts their ability to sign without resting a hand on the screen. 

## Conclusion

Patients who have been e-consented have expressed satisfaction in the ease of use and security of the software. Patients being unable to rest their hands on the screen is being examined. Newer tablets can ignore inputs other than the stylus.

The MS Register intends to use the software in additional centres to capture patient consent.

